# Central Bone Mineral Density Is Not a Reliable Surrogate for Assessing Suitable Bone Strength for Cementless Total Knee Arthroplasty

**DOI:** 10.3390/jcm14207384

**Published:** 2025-10-19

**Authors:** Dong Hwan Lee, Dai-Soon Kwak, Yong Deok Kim, Nicole Cho, In Jun Koh

**Affiliations:** 1 Department of Orthopaedic Surgery, Yeouido St. Mary’s Hospital, Seoul 07345, Republic of Korea; ldh850606@naver.com; 2 Department of Orthopaedic Surgery, College of Medicine, The Catholic University of Korea, Seoul 06591, Republic of Korea; seraph622@naver.com; 3 Catholic Institute for Applied Anatomy, Department of Anatomy, College of Medicine, The Catholic University of Korea, Seoul 06591, Republic of Korea; daisoon@catholic.ac.kr; 4 Joint Replacement Center, Eunpyeong St. Mary’s Hospital, Seoul 03312, Republic of Korea; 5 Hackensack Meridian School of Medicine, 123 Metro Blvd, Nutley, NJ 07100, USA; nicole.cho@hmhn.org

**Keywords:** dual-energy X-ray Absorptiometry, bone density, arthroplasty, replacement, knee, central bone mineral density, cementless

## Abstract

**Background/Objectives**: Central bone mineral density (cBMD) is widely utilized for assessing bone quality, but its reliability as a predictor of knee bone strength for cementless total knee arthroplasty (TKA) remains unclear. This study aimed to determine whether cBMD reliably estimates bone strength suitable for cementless fixation. **Methods**: 188 patients scheduled for TKA underwent preoperative cBMD assessment of the lumbar spine and femoral neck. During surgery, femoral bone specimens were collected for indentation tests. We compared distal femoral bone strength among osteoporosis classification groups (normal, osteopenia, osteoporosis) and examined the distribution of cementless suitable versus cemented mandatory cases with chi-square tests. ROC analysis evaluated cBMD’s diagnostic performance in predicting cementless TKA suitability, with AUC, sensitivity, and specificity calculated for both measurement sites. **Results**: No significant differences in distal femoral bone strength existed between osteopenia and osteoporosis groups (*p* = 0.845 for lumbar spine, *p* = 0.857 for femoral neck). Among patients with normal cBMD, 35.4% (lumbar spine) and 30.7% (femoral neck) were unsuitable for cementless TKA, whereas 30.8% and 45.0% of osteoporotic patients, respectively, had adequate bone strength for cementless fixation. The AUC values for predicting cementless suitability were 0.656 (lumbar spine) and 0.669 (femoral neck), with sensitivity and specificity below 0.75 for both measurements. **Conclusions**: Central BMD does not reliably represent distal femoral bone strength and demonstrates inadequate predictive capability for identifying appropriate candidates for cementless TKA in this predominantly Asian female cohort. Future multi-center, multi-ethnic studies are needed to enhance generalizability.

## 1. Introduction

Cemented fixation has traditionally dominated total knee arthroplasty (TKA) due to concerns over initial fixation stability associated with cementless approaches. However, the demographic change in patients undergoing TKA has markedly evolved in recent years [1,2]. The procedure is increasingly performed in younger, more active individuals and patients with obesity, leading to implant loosening emerging as a prominent cause for revision surgery [3,4,5,6]. Concurrently, advancements in manufacturing technology, particularly enhanced porous coating techniques, have revitalized interest in cementless TKA applications [7,8]. Despite this renewed focus, adequate bone quality remains the fundamental determinant of successful cementless TKA outcomes, yet a standardized method for preoperative assessment of suitable bone strength remains unclear in clinical practice.

Central bone mineral density (cBMD) assessed via Dual-energy X-ray Absorptiometry (DXA) is the predominant method employed for estimating approximate bone quality. However, contemporary research has produced conflicting evidence regarding its efficacy in reflecting peripheral joint bone quality. While some investigations report meaningful correlations between cBMD and peripheral bone properties, others report substantial regional variations or entirely discordant relationships [9,10,11]. Several studies specifically question whether central measurements adequately represent bone quality in anatomically distant sites such as the knee [12,13,14]. This inconsistency raises significant concerns about the validity of cBMD as a surrogate marker for peripheral joint bone strength assessment, particularly when determining candidates for cementless TKA. This discrepancy may be attributed to the fundamental biomechanical and metabolic differences between the axial and appendicular skeletons. The distal femur experiences multidirectional mechanical loading and higher remodeling activity [15]. Moreover, its trabecular bone composition and architecture differ substantially from those of the spine or femoral neck [16], potentially explaining why central BMD fails to represent peripheral joint bone strength.

Therefore, this study aimed to evaluate whether cBMD reliably predicts bone strength suitable for cementless TKA by examining two specific objectives: (1) to analyze differences in distal femoral bone strength across osteoporosis classification groups (normal, osteopenia, osteoporosis) as determined by lumbar total and femoral neck cBMD measurements, and to characterize the distribution of cementless-suitable candidates within each classification group; and (2) to assess the diagnostic accuracy of cBMD in predicting bone strength appropriate for cementless fixation.

## 2. Materials and Methods

### 2.1. Study Population

This prospective study included 188 consecutive patients who underwent primary TKA between May 2022 and May 2024. The surgical intervention employed posterior-stabilized designs from the Triathlon^®^ knee system (Stryker Inc., Mahwah, NJ, USA) in all cases. The study protocol received approval from our Institutional Review Board (PC22OISI0068), and written informed consent was obtained from each participant prior to enrollment. Although we planned to exclude non-consenting individuals, universal consent was achieved among eligible patients. All demographic and clinical data were prospectively collected from the institutional electronic medical record system and preoperative imaging database. Variables abstracted for analysis included age, sex, weight, height, body mass index (BMI), and DXA measurements of the lumbar spine and femoral neck. Two investigators independently verified all data entries to ensure accuracy and completeness.

### 2.2. Central BMD Assessment

Preoperative bone density evaluation was performed approximately three months before surgery using a Horizon^®^ DXA system (Hologic, Inc., Bedford, MA, USA). We examined two anatomical regions: the total lumbar spine and the femoral neck. Classification by lumbar spine measurements revealed 60% with normal density, 33% with osteopenia, and 7% with osteoporosis. Femoral neck assessment yielded a different distribution: 40% normal, 50% osteopenia, and 10% osteoporosis.

### 2.3. Clinical Fixation Type Selection Protocol

In clinical practice, the fixation type, whether cementless or cemented, was determined based on two main criteria previously established by the authors. First, preoperative Hounsfield unit (HU) values were obtained from conventional CT scans and compared with previously published osteoporosis cutoff values for the distal femur and proximal tibia [17]. Second, intraoperative visual assessment by the operating surgeon at the time of bone resection. Specifically, the surgeon evaluated the resected bone surface, focusing on the cortical contour and the size and density of the exposed trabecular pores. These clinical fixation decisions were made completely independently of the present study.

In contrast, within this study, the classification of each case as “cementless suitable” or “cemented mandatory” was performed postoperatively according to the predefined research protocol, based on the comparison between the estimated withstanding strength (EWS) and the minimum required strength (MRS). Although the surgeon was aware of the preoperative cBMD values, these data were not considered in the clinical decision-making process for implant fixation. All patients had a minimum follow-up of two years, with no revision cases observed. No patients experienced implant loosening, and no radiolucency was observed in any patient.

### 2.4. Bone Strength Determination

**Figure 1:**
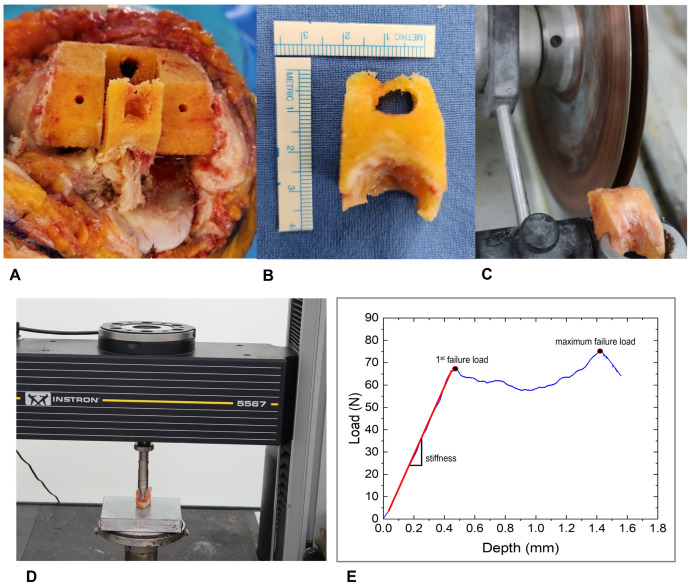
(**A**) Intraoperative harvesting of femoral box bone during TKA procedure. (**B**) The harvested box bone specimen. (**C**) Box bone specimen sectioned to 6 mm thickness for indentation testing. (**D**) Application of controlled pressure using a 6 mm diameter cylindrical flat-ended punch. (**E**) Data acquisition system recording mechanical parameters.

Mechanical strength was characterized via indentation testing, implementing methodologies delineated in prior studies [13,18,19]. During the box preparation of posterior-stabilized TKA, femoral box bone specimens were harvested (Figure 1). These specimens were preserved at −70 °C to maintain structural integrity until laboratory evaluation. Before testing, each specimen underwent a standardized preparation protocol. After gradual thawing to room temperature, we used a precision linear saw (IsoMet 5000; Buehler; Lake Bluff, IL, USA) equipped with paired diamond blades to create uniform 6 mm thick samples. These standardized specimens were then secured in a mechanical testing apparatus (Instron 5567; Norwood, MA, USA) for mechanical assessment. The mechanical testing involved applying controlled pressure using a cylindrical flat-ended punch (6 mm diameter) that created a consistent contact surface of 28.3 mm^2^. Initial contact was established with a minimal 2 N preload to ensure proper positioning. We then applied continuous pressure with a crosshead speed of 1.0 mm per minute, penetrating to a depth of 2 mm while recording the bone’s resistance. Throughout this process, the testing system captured force and displacement measurements at 30 Hz intervals, with data processing performed using specialized software (Instron Bluehill v4.23) (Figure 1).

Our primary measurement of interest, first failure load, represents the point where the specimen initially yields under pressure, visible as the first departure from linearity on the force-displacement graph. Across our sample, this critical value averaged 59.8 ± 38.1 N (range: 7.2–202.5 N). The average compressive displacement at this first peak force was 0.9 ± 0.3 mm (range: 0.3–1.8 mm). We also recorded the maximal force, which averaged 81.5 ± 42.0 N (range: 14.6–244.0 N) at a mean displacement of 1.8 ± 0.3 mm. The overall stiffness of the specimens was 111.6 ± 80.5 N/mm (range: 12.0–533.8 N/mm) (Table 1).

### 2.5. Defining Strength-Based Criteria for Cementless TKA

**Figure 2:**
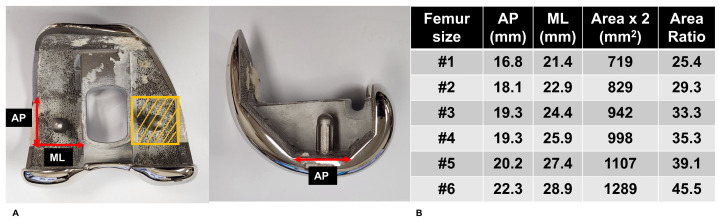
(**A**) Determination of total distal femoral component surface area by doubling the product of anterior–posterior and medio-lateral distances. (**B**) Actual distal surface area (mm^2^) according to implant size and its ratio to the indenter surface area (28.3 mm^2^).

To determine cementless TKA suitability, we established two key metrics. These metrics were specifically defined by the authors for this study. First, the Minimum Required Strength (MRS) was determined by multiplying each patient’s body weight by 2.5. This threshold was selected based on previous biomechanical research showing that routine activities following TKA can generate knee joint forces reaching this magnitude [20,21]. Second, the Estimated Withstanding Strength (EWS) for each specimen was derived by scaling the measured first failure load based on the proportional relationship between the femoral component distal cutting surface and the indenter cross-section of 28.3 mm^2^ (Figure 2). By comparing these values, we categorized patients into two groups. We designated participants as “cementless suitable” when EWS exceeded MRS, and as “cemented mandatory” when EWS fell below MRS. Through this classification, we analyzed whether cBMD could serve as an appropriate tool for identifying suitable candidates for cementless TKA. It should be noted that this MRS represents a minimum threshold for axial compression during routine gait activities and does not account for more complex loading scenarios. Future work incorporating multi-axial loading conditions, patient activity levels, and surgical factors is needed to refine the biomechanical criteria for cementless TKA candidacy.

### 2.6. Statistical Analysis

We performed all statistical computations using SPSS version 21 (IBM Corp., Armonk, NY, USA), with statistical significance defined as *p* < 0.05. One-way analysis of variance examined EWS differences across the three osteoporosis categories (normal, osteopenia, osteoporosis). Following the overall F-test, we applied Tukey’s HSD method for post hoc pairwise testing to address multiple comparison issues. For each osteoporosis group, the distribution of cementless suitable versus cemented mandatory cases was assessed using chi-square tests. Receiver operating characteristic (ROC) analysis was performed to evaluate how well central bone mineral density predicts sufficient bone strength in patients undergoing cementless fixation. The area under the curve (AUC), optimal threshold values, sensitivity, specificity, positive predictive value (PPV), and negative predictive value (NPV) were calculated for both lumbar total and femoral neck measurements. AUC values were interpreted according to established criteria: excellent (0.9–1.0), good (0.8–0.9), fair (0.7–0.8), poor (0.6–0.7), and fail (0.5–0.6).

## 3. Results

Table 2 presents the demographic and osteoporosis information of study participants. Participants averaged 68.1 years (SD 5.4) in age (53 to 86 years), with females comprising 82% of the cohort. Mean height measured 155.5 cm (SD 7.0), while mean body weight was 65.7 kg (SD 9.6), yielding an average BMI of 27.2 kg/m^2^ (SD 3.4). DXA measurements showed lumbar spine T-scores averaging −0.5 ± 1.5 (−3.6 to 4.9) and femoral neck T-scores averaging −1.2 ± 1.1 (−3.5 to 2.8).

**Figure 3:**
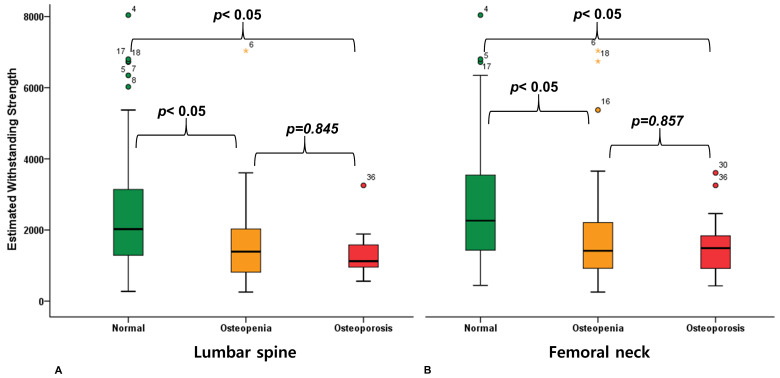
Comparison of estimated withstanding strength (EWS) values among normal, osteopenia, and osteoporosis groups based on (**A**) lumbar spine and (**B**) femoral neck cBMD classifications. ANOVA revealed no significant differences between osteopenia and osteoporosis groups for either measurement site (*p* = 0.845 for lumbar spine, *p* = 0.857 for femoral neck).

**Figure 4:**
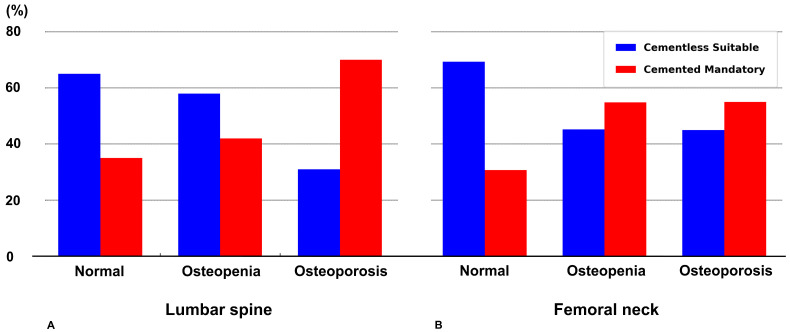
Distribution of cementless suitable versus cemented mandatory cases according to (**A**) lumbar spine and (**B**) femoral neck cBMD classifications. For lumbar spine cBMD, 35.4% of individuals categorized as “normal” were unsuitable for cementless TKA, while 30.8% of those classified as “osteoporosis” demonstrated adequate bone strength for cementless fixation. For femoral neck cBMD, 30.7% of individuals categorized as “normal” were unsuitable for cementless TKA, whereas 45.0% of those classified as “osteoporosis” had adequate bone strength for cementless fixation.

No significant differences in EWS existed between osteopenia and osteoporosis groups for both lumbar spine and femoral neck cBMD classifications. ANOVA demonstrated a significant omnibus difference in distal femoral bone strength across groups for both lumbar spine (F = 8.812, *p* < 0.001) and femoral neck (F = 11.873, *p* < 0.001). No significant difference in distal femoral bone strength was observed between osteopenia and osteoporosis groups using Tukey’s HSD test, based on either lumbar spine measurements (*p* = 0.845) or femoral neck measurements (*p* = 0.857) (Figure 3). Furthermore, 35.4% of individuals categorized as “normal” by lumbar spine cBMD were unsuitable for cementless TKA, while 30.8% of those classified as “osteoporosis” demonstrated adequate bone strength for cementless fixation. Similarly, 30.7% of individuals categorized as “normal” by femoral neck cBMD were unsuitable for cementless TKA, whereas 45.0% of those classified as “osteoporosis” had adequate bone strength for cementless fixation. (Figure 4).

**Figure 5:**
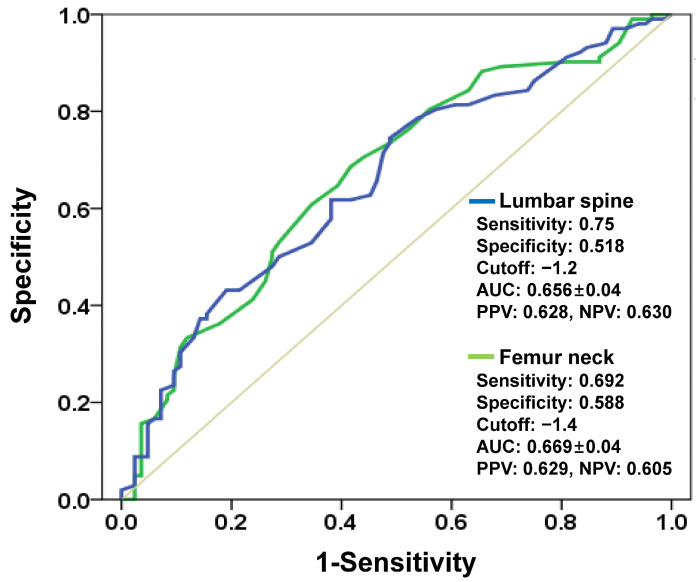
ROC curve analysis of lumbar spine and femoral neck cBMD for discriminating sufficient bone strength in patients undergoing cementless fixation. For the lumbar spine, the AUC was 0.656 ± 0.04, sensitivity 75.0%, specificity 51.8%, PPV 62.8%, and NPV 63%; for the femoral neck, the AUC was 0.669 ± 0.04, sensitivity 69.2%, specificity 58.8%, PPV 62.9%, and NPV 60.5%.

cBMD showed poor diagnostic accuracy in predicting bone strength adequate for cementless fixation. ROC curve analysis demonstrated AUC values of 0.656 for lumbar spine and 0.669 for femoral neck, both of which fall within the poor diagnostic category (Figure 5). Sensitivity and specificity were each below 0.75, suggesting a substantial risk of both false-positive and false-negative assessments when using cBMD alone.

## 4. Discussion

Adequate local bone strength is essential for the success of cementless TKA outcomes. However, no established gold standard exists for its preoperative assessment. Although cBMD is routinely employed for general bone quality evaluation, its capability to predict knee-specific bone strength and appropriateness for cementless implantation remains uncertain. Our investigation sought to determine whether conventional cBMD measurements could effectively predict distal femoral bone strength and identify suitable candidates for cementless fixation.

These results provide exploratory evidence that cBMD does not accurately predict actual distal femoral bone strength. In this study, distal femoral bone strength did not significantly differ between osteopenia and osteoporosis groups (*p*-values of 0.845 and 0.857 for the lumbar spine and femoral neck, respectively). The absence of marked differences in distal femoral bone strength between osteopenia and osteoporosis groups suggests that conventional osteoporosis classification based on cBMD measurements does not reliably translate to peripheral joint bone quality. This discordance aligns with previous investigations highlighting the limitations of cBMD measurements in reflecting peripheral bone characteristics. Some studies have reported that peripheral bone quality does not correlate with cBMD depending on the patients’ medical characteristics or age group, and correlations may also vary according to the anatomical region around the joint [9,10,12]. These observations reinforce the concept that regional variations in bone quality may not be adequately captured by centralized measurement techniques, thus limiting the applicability of cBMD in peripheral joint assessment.

The diagnostic performance of cBMD in identifying suitable candidates for cementless TKA proved inadequate in our analysis. Although cBMD has traditionally been utilized for osteoporosis screening, its diagnostic performance in predicting distal femoral bone strength appears to be limited in this study. The AUC values for lumbar total and femur neck were 0.656 and 0.669, respectively, which fall within the “poor” diagnostic category. Moreover, the optimal sensitivity and specificity for each measure were both below 0.75, indicating a relatively high rate of false positives and false negatives. These findings suggest that central BMD measurements may not adequately reflect the local bone quality required for procedures such as cementless TKA. Our results indicate the need for peripheral joint-specific assessment tools to accurately evaluate bone quality parameters for the use of cementless TKA. Recent studies have demonstrated that plain radiographs, when analyzed using grayscale histogram analysis or deep learning algorithms, can provide useful information about local bone quality and demonstrate promising correlations with DXA measurements [22,23,24]. However, these approaches require further validation and standardization before they can be reliably adopted for preoperative bone strength assessment in cementless TKA. Measuring BMD using HU from conventional CT has proven to be a valuable assessment tool, demonstrating strong correlations with bone strength [25,26,27,28]. Furthermore, quantitative CT (QCT) enables more precise evaluation of bone quality through calibrated volumetric BMD measurements [29,30,31]. Recent research reports that dual-energy CT technology provides even greater accuracy in volumetric BMD quantification by distinguishing bone marrow fat tissue and applying appropriate corrections [32,33]. These CT-based bone quality assessment methods may offer more reliable evaluation of bone quality relevant to cementless TKA candidacy.

There are several limitations in this study. First, our study cohort was relatively homogeneous, consisting exclusively of Asian patients and predominantly females (82%), which reflects the typical demographic profile of primary TKA patients in our region [34]. This demographic homogeneity limits the broader applicability of our findings. It is well-established that BMD and bone strength differ significantly by ethnicity and gender [35,36]. Furthermore, previous studies have also documented marked ethnic disparities in lower limb alignment characteristics [37]. Therefore, future research with a broader, more diverse demographic composition, including varying ethnicities and balanced gender representation, is needed to enhance the applicability of these results globally.

Second, our EWS calculation utilized the distal femoral resection surface cross-sectional area, yet mechanical testing was conducted exclusively on box bone specimens. In other words, the EWS calculation was based on the bone strength of the box bone rather than that of the distal femoral cutting surface. This approach was necessary because obtaining bone specimens of adequate thickness and uniform quality from the distal femoral cutting surface was not feasible. However, given that the box bone typically demonstrates lower strength than the distal femoral resection surface, the EWS provides a more cautious estimate. Therefore, this discrepancy should not increase the risk of cementless TKA failure. Nevertheless, EWS values should be interpreted within the anatomical and methodological constraints of this study. Although the box bone area generally has lower strength, actual experimental specimens do not always follow this general pattern [38], potentially due to localized variations in trabecular density and subchondral bone quality within the distal femur [15,16]. Therefore, the discrepancy between the area where indentation testing was performed and the area used for EWS calculation can introduce bias, representing a distinct limitation of this study. Furthermore, we only evaluated the distal femur and could not assess the tibial bone, which is notably more prone to subsidence in cementless TKA. Tibial bone specimens obtained after resection are relatively thin and non-uniform, with bone strength varying significantly according to resection level and proximity to subchondral bone [39,40]. This makes it challenging to obtain consistent quality specimens for experimental testing. However, considering that tibial bone quality is generally regarded as the most important factor in cementless TKA selection, the fact that we could not evaluate tibial bone strength represents a significant limitation of this study. This was an experimental study designed to evaluate the diagnostic value of cBMD in screening cementless TKA suitability, and despite analyzing only femoral box bone, we were able to demonstrate its poor diagnostic value. However, to establish screening criteria for cementless TKA suitability, evaluation of tibial bone quality, where failures occur more frequently, is essential. To emphasize once again, this study has clear limitations in that only the femoral side was examined. Future research should assess tibial bone strength and investigate how bone strength changes with the degree of tibial bone resection to enable more precise cementless TKA selection.

Third, we set the MRS by multiplying individual body weight by 2.5, drawing from previous research that identified this level as sufficient to withstand the forces encountered during routine activities like walking [20,21]. This simplified approach does not account for all possible loading scenarios, and actual mechanical demands may vary considerably depending on surgical technique, limb alignment, soft tissue balance, joint stability, and patient activity levels. While this approach was appropriate for the primary objective of evaluating whether cBMD correlates with local bone strength at the distal femur, a more comprehensive biomechanical simulation or prospective clinical validation would be needed to fully explore the dynamic variability of MRS in individual cases.

Finally, we assessed only uniaxial compressive failure limits, which represents an important limitation of our study methodology. In actual cementless TKA implant failure mechanisms, micromotion, interface shear, bending, and torsional forces play substantial roles in determining fixation success, which were not addressed in our evaluation. These complex multiaxial loading conditions could not be adequately captured through our indentation testing methodology, representing a clear limitation when assessing overall cementless TKA suitability. However, we believe that even with this constraint to uniaxial compressive testing alone, our results provide meaningful exploratory evidence that cBMD is inadequate for evaluating cementless TKA candidacy. This finding highlights the need for additional methods to comprehensively assess peripheral bone quality. In future research, modeling studies integrating bone quality parameters with clinical and demographic factors could help predict the optimal fixation type and refine preoperative selection algorithms in TKA. Such predictive approaches may enhance the accuracy of fixation planning and contribute to personalized arthroplasty strategies, as suggested by recent modeling frameworks in orthopedic research [41]. Despite these limitations, our investigation provides meaningful evidence challenging the reliability of cBMD as a surrogate marker for assessing distal femoral bone strength suitability in cementless TKA candidates.

## 5. Conclusions

Central BMD does not reliably represent distal femoral bone strength and demonstrates inadequate predictive capability for identifying appropriate candidates for cementless TKA. This suggests the need for more advanced imaging-based approaches—such as HU from conventional CT and volumetric CT-based assessment (including QCT and dual-energy CT)—to accurately assess peripheral joint bone quality when determining suitability for cementless TKA. Given the predominantly Asian female composition of our cohort and the limitation to femoral bone assessment only, future multi-center, multi-ethnic studies incorporating tibial bone strength evaluation are needed to enhance generalizability and provide comprehensive assessment of cementless TKA candidacy.

## Figures and Tables

**Table 1:** Mechanical characteristics of harvested bone specimens *.

Parameters	Values (n = 188)
First failure load (N)	59.8 ± 38.1 (7.2~202.5)
Displacement at first failure load (mm)	0.9 ± 0.3 (0.3~1.8)
Maximum failure load (N)	81.5 ± 42.0 (14.6~244.0)
Displacement at maximum failure load (mm)	1.8 ± 0.3 (0.4~2.0)
Stiffness (N/mm)	111.6 ± 80.5 (12.0~533.8)

* Values are expressed as mean ± standard deviation (range: minimum to maximum).

**Table 2:** Demographics and osteoporosis information.

Parameters	Values (n = 188)	
Demographic information *		
Age (year)	68.1 ± 5.4 (53~86)	
Gender (women) †	154 (82)	
Height (cm)	155.5 ± 7.0 (143.1~177.2)	
Weight (kg)	65.7 ± 9.6 (45.4~94.3)	
BMI (kg/m^2^)	27.2 ± 3.4 (19.6~36.5)	
Osteoporosis information		
	Lumbar spine	Femoral neck
DXA (T-score) *	−0.5 ± 1.5 (−3.6~4.9)	−1.2 ± 1.1 (−3.5~2.8)
Prevalence †		
Normal (T score > −1.0)	113 (60)	75 (40)
Osteopenia (−1.0 ≤ T score ≤ −2.5)	62 (33)	93 (50)
Osteoporosis (T score < −2.5)	13 (7)	20 (10)

* Values shown as mean ± standard deviation with range (minimum to maximum); † Patient counts displayed with percentage in parentheses; BMI = body mass index; DXA = Dual-energy X-ray absorptiometry.

## Data Availability

The data that support the findings of this study are available from the corresponding author upon reasonable request.

## Abbreviations

The following abbreviations are used in this manuscript:

TKA	Total Knee Arthroplasty
cBMD	Central Bone Mineral Density
DXA	Dual-energy X-ray Absorptiometry
BMI	Body Mass Index
HU	Hounsfield Unit
MRS	Minimum Required Strength
EWS	Estimated Withstanding Strength
AUC	Area Under the Curve
ANOVA	Analysis of Variance
HSD	Honestly Significant Difference
